# Association of the *IL-1RN* variable number of tandem repeat polymorphism and *Helicobacter pylori* infection: A meta-analysis

**DOI:** 10.1371/journal.pone.0175052

**Published:** 2017-04-06

**Authors:** Jinhua Zhang, Xudong Sun, Jiemin Wang, Fuhua Zhang, Xiaohua Li, Jian Han

**Affiliations:** 1 Department of Gastroenterology, Second Hospital of Gansu Province, Lanzhou, China; 2 Department of Medicine, School of Second Clinical Medicine, Northwest University for Nationalities, Lanzhou, China; 3 Department of Pathogenic Biology, School of Basic Medical Sciences, Lanzhou University, Lanzhou, China; 4 Department of Gastroenterology, Liangzhou Hospital, Wuwei, China; Duke Cancer Institute, UNITED STATES

## Abstract

The aim of this study was to clarify the association of *IL-1RN* variable number of tandem repeat (VNTR) polymorphism and *H*. *pylori* infection. We performed a meta-analysis of studies retrieved by systematic searches of Pubmed, Embase and the Cochrane Library. Data were analyzed with STATA 13.1 using pooled odds ratios (ORs) with 95% confidence intervals (CIs). A total of 18 studies were included in our meta-analysis, and *IL-1RN* VNTR was found to be significantly associated with *H*. *pylori* infection in the comparisons of 22+2L *vs*. LL (OR = 1.17, 95% CI = 1.02–1.33) and 2 allele *vs*. L allele (OR = 1.18, 95% CI = 1.00–1.40). Stratified analyses on study designs and ethnicities were also conducted. *IL-1RN* VNTR was positively correlated with *H*. *pylori* infection in Asian subgroup and Hospital-Based subgroup (i.e., study samples obtained from hospital inpatients). In conclusion, our study demonstrated that *IL-1RN* VNTR polymorphism might increase the risk of *H*. *pylori* infection, especially in Asians.

## Introduction

*Helicobacter pylori* is a pathogen that was discovered by Warren and Marshall in 1983 [1], and is thought to be involved in gastritis, peptic ulcers, gastric cancer, and mucosa-associated lymphoid tissue (MALT) lymphoma [2–4]. More than half of the world’s population is infected with *H*. *pylori*. Multiple factors influence the outcomes of *H*. *pylori* infection. Host genetic factors, interacting with *H*. *pylori* virulence (VacA, CagA *etc*.), and environmental factors (high salt intake and nitrate consumption, *etc*.), are involved in the pathogenesis of gastric cancer [5–7]. *H*. *pylori* infection elicits adaptive and innate immune responses in the gastric mucosa that produce significant inflammation [6]. *H*. *pylori* can stimulates host secretion of cytokines including interleukin (IL)-1, -2, -4, -8, -10, -1 receptor antagonist (rα), tumor necrosis factor (TNF)-α and others that may contribute to persistent infection [6, 8–10]. Host genetic polymorphisms of several cytokine genes (e.g., *IL-1B*-511*T, IL-1-RN*2, *IL-10*-1082/-819/-592, *TNF*-A-308*A, and *IL-8*-251*A), innate immune response gene (*TLR4*+896*G), HLA (DQA1*03:01, DQA1*04:01, and DQB1*05:01:01) are involved in all stages of the neoplastic process in gastric carcinoma [6, 11–16]. Previous reports of the relationship of host genetic polymorphisms and *H*. *pylori* susceptibility are inconsistent. To begin to address these inconsistencies, we previously conducted two meta-analysis of the relationship between host *IL1B* -31C > T and *TNFA* gene polymorphisms and *H*. *pylori* infection [9, 10]. This meta-analysis focuses on the association of host *IL-1RN* variable number of tandem repeat (VNTR) polymorphism and *H*. *pylori* infection.

The *IL-1* genes cluster is located on the long arm of human chromosome 2, comprising *IL-1A*, *IL-1B* and *IL-1RN* [17]. *IL-1RN* encodes IL-1rα, which is the endogenous receptor antagonist of IL-1α and IL-1β. A penta-allelic 86-bp VNTR polymorphism is located in intron 2 of the *IL-1RN* gene. Alleles 1–5 contain 4 repeats, 2 repeats, 5 repeats, 3 repeats, and 6 repeats, respectively. The repeats can be divided into a long allele (*IL-1RN* *L, including alleles 1, 3, 4 and 5) and a short allele (*IL-1RN* *2, including allele 2) [18]. LL and 22 are homozygous genotypes, while 2L is heterozygous genotype. Some studies reported that *IL-1RN* VNTR polymorphism is associated with the secretion of IL-1rα, which could influence *H*. *pylori* infection by antagonizing IL-1α and IL-1β [19, 20].

The association between *IL-1RN* VNTR polymorphism and *H*. *pylori* related-diseases has been extensively investigated [21–23]. One meta-analysis reported that the short genotype of *IL-1RN* VNTR significantly increases the risk of gastric cancer [24]; another paper found that *IL-1RN* VNTR has no association with duodenal ulcer [25]. A number of studies performed on *H*. *pylori* related-diseases have explored the association between *IL-1RN* VNTR polymorphism and *H*. *pylori* infection simultaneously, but their results have been inconsistent. Therefore, we performed this meta-analysis to explore and analyze these inconsistent results. This is the first meta-analysis that focused on clarifying the relationship between *IL-1RN* VNTR polymorphism and *H*. *pylori* infection.

## Materials and methods

### Search strategy

A systematic literature search of the Pubmed, Embase and Cochrane Library databases entries to August 2016 was conducted. The following search terms were used: (IL-1RN OR IL1RN OR interleukin-1RN) AND (polymorphism OR polymorphisms OR SNP) AND (*Helicobacter pylori* OR *H*. *pylori* OR HP). The search was limited to the English language publications with available full-text. The reference lists of retrieved papers were also examined to search for potentially relevant studies. We contacted authors requesting the full-text of their work if necessary. When more than one report of the same case series had been published, only the study with the largest sample size was included in the meta-analysis.

### Selection criteria

The inclusion criteria of our meta-analysis were (1) investigation of the association of *IL-1RN* VNTR polymorphism and *H*. *pylori* infection was evaluated; (2) case-control designed on unrelated individuals; (3) use of objective and clearly described methods for detecting *H*. *pylori* infection; and (4) reporting of genotype data sufficient to calculate odds ratios (ORs) with 95% confidence intervals (CIs).

### Data extraction and quality appraisal

The authors; year of publication; country; ethnicity of participants; study design; number of cases and controls; methods of detecting *H*. *pylori* infection and distribution of polymorphism were extracted from each article. We evaluated study quality with the Newcastle-Ottawa scale (NOS) [26], which adopts three main criteria: selection of cases and controls; comparability of cases and controls; and exposure to risk factors. NOS scores were ranged from 0 to 9 stars. Articles with a final score 7 or more were considered to be of high quality, whereas those with a final score 5 or less were considered of low quality. Two authors (JZ and XS) independently extracted the data and performed the quality appraisal. Any disagreements between these two authors were resolved by discussion with the other authors.

### Statistical analysis

All statistical analyses were carried out using STATA 13.1 (STATA Corp, College Station, TX, USA). The combined ORs and their corresponding 95% CIs were used to assess the strength of the association between *IL-1RN* VNTR polymorphism and *H*. *pylori* infection. The Q-test and I^2^ index were used to determine heterogeneity across studies, with *P* < 0.10 or I^2^ > 50% considered significant [27]. The ORs were pooled using a random effect model in the presence of significant heterogeneity; otherwise, a fixed effect model was used. Sensitivity analyses were conducted to identify the effect of each study on the combined results by omitting each one in turn. Subgroup analyses were conducted based on ethnicities (Asian or Non-Asian) and study designs (population-based (PB) or hospital-based (HB)). Publication bias was evaluated by Begg’s funnel plots and Egger’s plots, with a significance of 0.05. Hardy-Weinberg equilibrium (HWE) was calculated by the χ^2^-square test.

## Results

### Study characteristics

**Fig 1:**
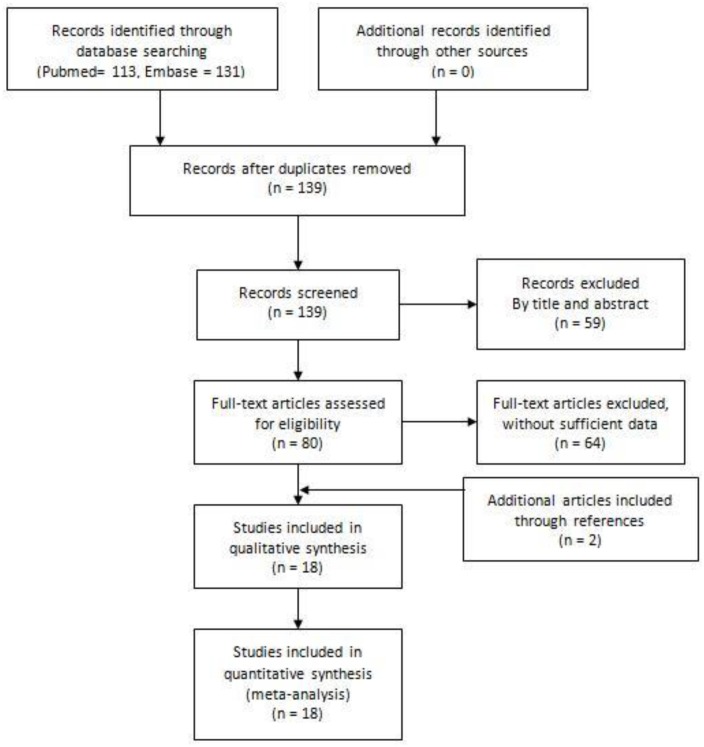
The study selection process of the meta-analysis.

The study selection process is shown in Fig 1. A total of 139 articles were retrieved in the initial search. 59 articles were excluded after screening the titles and abstracts, and 64 articles were excluded after reading the full text. Two articles were added after scanning references lists. In total, 18 articles were included in our meta-analysis [18, 19, 28–43]. Table 1 lists the major characteristics of the included studies. Of the included studies, 11 were performed in Asians, 1 was in Europeans, 1 was in Africans and 5 were in mixed-ethnicity populations.

**Table 1:** Main characteristics of studies included in meta-analysis.

Author	Year	Country	Ethnicity	Study design	No. of *Hp*(+)	No. of *Hp*(-)	Detection of *Hp*	NOS(score)	HWE(P)
Hamajima	2001	Japan	Asian	HB	151	90	ELISA	5	0.743
Furuta	2002	Japan	Asian	PB	579	227	RUT, HE, BC	7	0.753
Uno	2002	Brazil	Asian	PB	457	490	ELISA	6	0.947
Gatti	2004	Brazil	Mixed	HB	37	75	RUT	9	0.131
Vilaichone	2005	Thailand	Asian	HB	101	29	ELISA,RUT,HE,BC,PCR	6	0.534
Chakravorty	2006	India	Asian	HB	153	157	RUT,HE,BC	6	0.106
Kim	2006	Korea	Asian	PB	1174	186	ELISA, RUT, HE, BC	6	0.267
Li	2007	China	Asian	HB	374	289	ELISA	8	0.288
Gonzalez	2009	Spain	Europeans	HB	57	24	RUT, HE	5	0.759
Kumar	2009	India	Asian	HB	167	79	MI	7	0.822
Queiroz	2009	Brazil	Mixed	PB	370	170	ELISA	7	0.004
He	2011	China	Asian	HB	491	409	ELISA	7	0.529
Kang	2012	Korea	Asian	HB	284	116	RUT,HE	7	0.592
Kimang’a	2012	Kenya	African	HB	151	119	RUT,HE,HpSAT,PCR	4	0.000
Santos	2012	Brazil	Mixed	HB	174	26	RUT,HE,PCR	5	0.068
Queiroz	2013	Brazil	Mixed	HB	46	78	RUT,UBT,HE,BC	6	0.059
Kulmambetova	2014	Kazakhstan	Asian	HB	145	389	HE	6	0.000
Drici	2016	Algeria	Mixed	HB	79	32	ELISA,HE	7	0.706

*Hp*: *H*. *pylori;* +: positive; -: negative; PB: population-based; HB: hospital-based; ELISA: enzyme-linked immunosorbent assay; RUT: rapid urease test; HE: histological examination; BC: bacteria culture; UBT: urease breath test; HpSAT: *Helicobacter pylori* stool antigen test; PCR: polymerase chain reaction; MI: molecular identification.

### Meta-analysis results

**Fig 2:**
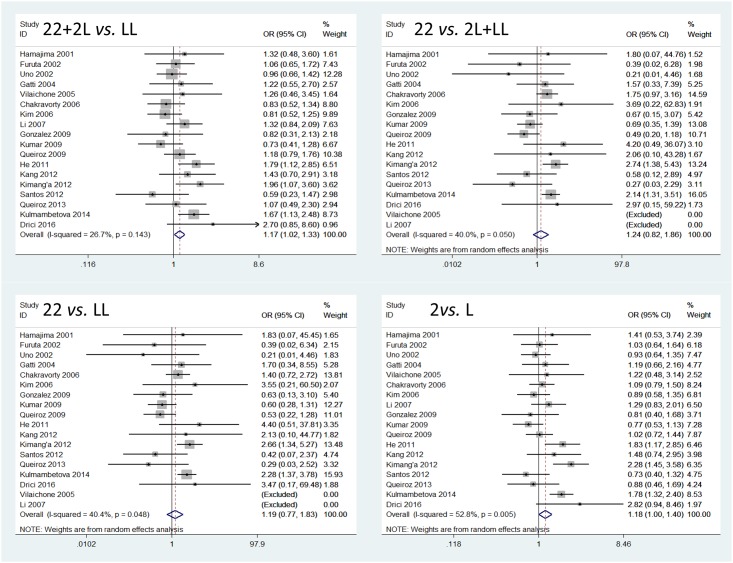
Forest plots of *IL-1RN* VNTR polymorphism and *H*. *pylori* infection for all genetic models.

*IL-1RN* VNTR polymorphism was significantly associated with *H*. *pylori* infection in the comparisons of 22+2L *vs*. LL and 2 allele *vs*. L allele (22+2L *vs*. LL, OR = 1.17, 95% CI = 1.02–1.33; 22 *vs*. 2L+LL, OR = 1.24, 95% CI = 0.82–1.86; 22 *vs*. LL, OR = 1.19, 95% CI = 0.77–1.83; 2 allele *vs*. L allele, OR = 1.18, 95% CI = 1.00–1.40; Fig 2).

Our subgroup analysis on ethnicity showed that in Asian populations, *IL-1RN* VNTR significantly increased the risk of *H*. *pylori* infection in the comparisons of 22 *vs*. 2L+LL and 22 *vs*. LL. When the analysis was stratified by study design, *IL-1RN* VNTR was significantly correlated with *H*. *pylori* infection in the comparisons of 22+2L *vs*. LL, 22 *vs*. 2L+LL and 2 allele *vs*. L allele for HB subgroup, but not for PB subgroup. The meta-analysis results are summarized in Table 2.

**Table 2:** Meta-analysis of the association between *IL-1RN* VNTR polymorphism and *H*. *pylori* infection.

Study Group	Study(n)	22+2L *vs*. LL		22 *vs*. 2L+LL		22 *vs*. LL		2 allele *vs*. L allele	
		OR	95% CI	OR	95% CI	OR	95% CI	OR	95% CI
Overall	18	**1.17**	**(1.02–1.33)**	1.24	(0.82–1.86)	1.19	(0.77–1.83)	**1.18**	**(1.00–1.40)**
Asian	11	1.14	(0.98–1.33)	**1.54**	**(1.13–2.12)**	**1.48**	**(1.06–2.08)**	1.18	(0.97–1.44)
PB	4	1.00	(0.81–1.23)	0.60	(0.29–1.22)	0.62	(0.30–1.29)	0.97	(0.79–1.18)
HB	14	**1.28**	**(1.09–1.51)**	**1.60**	**(1.22–2.11)**	1.40	(0.90–2.17)	**1.28**	**(1.03–1.58)**

Significant results were shown in bold.

### Heterogeneity and sensitivity analysis

Significant heterogeneity among studies existed in the comparison of 22 *vs*. 2L+LL, 22 *vs*. LL and 2 allele *vs*. L allele. A study by Queiroz *et al*. [39] was found to be the source of heterogeneity by omitting each study in turn. When sensitivity analyses were conducted, the pooled ORs were not significantly altered.

### Publication bias

**Fig 3:**
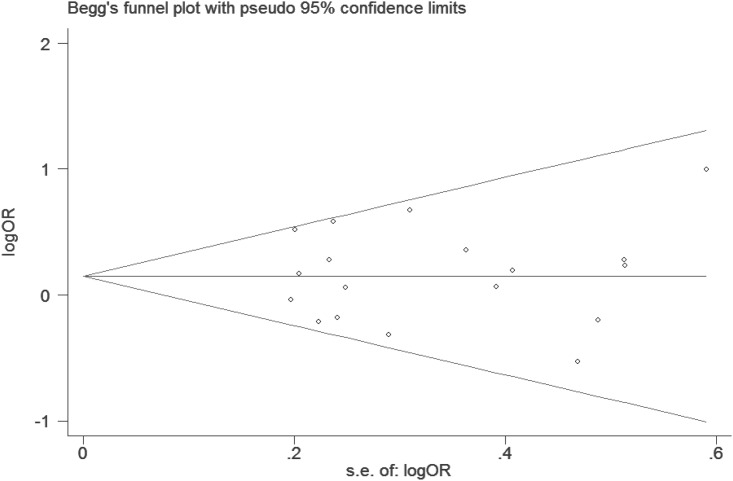
Begg’s funnel plot of studies included in the meta-analysis. s.e.: standard error.

Funnel plots are commonly used to evaluate publication bias, with asymmetry indicating possible publication bias. Begg’s funnel plot was performed in our meta-analysis, and the plot showed a nearly symmetrical distribution for the comparison of 22+2L *vs*. LL (Fig 3), Publication bias was not indicated by either Begg’s or Egger’s tests (22+2L *vs*. LL, Begg’s test *P* = 0.76, Egger’s test *P* = 0.93; 22 *vs*. 2L+LL, Begg’s test *P* = 0.75, Egger’s test *P* = 0.30; 22 *vs*. LL, Begg’s test *P* = 1.00, Egger’s test *P* = 0.30; and 2 allele *vs*. L allele, Begg’s test *P* = 0.60, Egger’s test *P* = 0.94).

## Discussion

Previous studies demonstrated that polymorphisms of some host cytokine genes such as IL-1β, IL-8 *et al*. are correlated with *H*. *pylori* infection related-diseases [17, 44, 45]. IL-1rα can influence IL-1β levels, and some studies have focused on the relationship between *IL-1RN* VNTR polymorphism and *H*. *pylori* infection related-diseases [46, 47]. Others have investigated the association between *IL-1RN* VNTR polymorphism and *H*. *pylori* infection. Because the conclusions of the available studies were not consistent [9, 48], we performed this meta-analysis to investigate the role of *IL-1RN* VNTR polymorphism on the risk for *H*. *pylori* infection.

We found that *IL-1RN* VNTR polymorphism has significant association with *H*. *pylori* infection, especially in Asians. These results differ from the findings of a genome wide association study in Europeans [49]. Based on including studies of our meta-analysis, we found that the frequency of *IL-1RN* *2 in Asians is lower than that in other ethnicities. Different ethnicities with different genetic background and living habits might be the source of discrepancy. Genetic differences of *H*. *pylori* (*cagA* positive or negative) might also influence the association of host *IL-1RN* VNTR polymorphism and *H*. *pylori* infection. Nearly all *H*. *pylori* in East Asian, but not Western, are *cagA* positive strains [50]. Stratified analysis revealed that *IL-1RN* VNTR polymorphism increased the risk of *H*. *pylori* infection for HB subgroups. This indicates that *IL-1RN* VNTR polymorphism may be associated with outcomes of *H*. *pylori* infection and warrants further investigation. Studies included in meta-analyses frequently differ to an extent that leads to significant heterogeneity. In this analysis, the heterogeneity decreased after excluding the study of Queiroz *et al*, which included 125 Brazilian children and adolescents undergoing gastrointestinal endoscopy. Specific ethnicity and age composition might be the source of heterogeneity. We used a random effects model when heterogeneity was detected among the evaluated studies.

*IL-1RN* gene encodes the cytokine IL-1rα, which is an endogenous receptor antagonist of IL-1β. Previous studies indicated that carriers of the IL-1RN*2 allele had significantly higher expression of the IL-1β than carriers of other genotypes had [51, 52]. A high level of IL-1β in the gastric mucosa can inhibit the function of gastrin-stimulated enterochromaffin cells and parietal cells, which leads to low histamine concentration and decreased gastric acid secretion [53, 54]. In addition, IL-1β can also amplify immune responses by activating neutrophils, T cells and B cells [55]. The combined activity change from the decreased acid secretion and amplified immune responses may lead to tissue damage of the gastric mucosa, which can facilitate the colonization of *H*. *pylori* from the gastric antrum to the corpus [56]. This colonization can contribute to persistent *H*. *pylori* infection and increase the risk of developing atrophic gastritis and gastric cancer.

CagA is an important *H*. *pylori* virulence factor, and is associated with severe gastritis and gastric carcinoma [57, 58]. CagA-negative *H*. *pylori* is weakly pathogenic or nonpathogenic. Differences of the repeat sequences of the 3′ region of *cagA* have led to recognition of East Asian-type and Western-type CagA [5]. East Asian-type *cagA* strains have greater pathogenicity and posing an increased risk of peptic ulcer or gastric cancer than Western-type *cagA* strains. CagA can be inserted into gastric epithelial cells by the *cag* PAI-encoded type IV secretion system and perform virulence through phosphorylation-dependent and phosphorylation-independent manner. Src homology-2 domain-containing phosphatase 2 (SHP2) is an important intracellular target of CagA in phosphorylation-dependent pathway [5, 57, 59]. The difference of East Asian-type and Western-type CagA in pathogenicity may result from the higher binding affinity of East Asian-type CagA for SHP-2 by Glu-Pro-Ile-Tyr-Ala (EPIYA)-D segments than Western-type CagA, which binds to SHP-2 by EPIYA-C segments [50]. East Asian-type *cagA* strains primarily circulate in East Asia (e.g., China, Japan, and Korea). Although the *H*. *pylori cagA* genotype has a significantly wider geographical distribution, our analysis was not stratified by *H*. *pylori cagA* genotypes because only one of the 18 articles selected for analyses assayed host *IL-1RN* gene polymorphism and *H*. *pylori cagA* genotypes [42]. The investigators found that host *IL-1* polymorphism and the *H*. *pylori cagA* genotype influenced gastric mucosal cytokine levels in patients in Thailand [42].

This is the first meta-analysis that investigated the association between *IL-1RN* VNTR polymorphism and *H*. *pylori* infection across multiple studies. However, there were some limitations to our study. Most of included studies were performed on Asian populations, so further research with other ethnic populations is needed. We only chose the English literatures retrieved from databases of PubMed, Embase and Cochrane library, which might lead to bias on collecting literatures.

## Conclusion

Based on including studies of our meta-analysis, we concluded that *IL-1RN* VNTR*2 may increase the risk of *H*. *pylori* infection, especially in Asians. Our findings provide insights into the role of *IL-1RN* VNTR polymorphism in *H*. *pylori* infection and related diseases. Further studies with larger sample sizes and various ethnicities are required to validate these results.

## Supporting information

S1 File PRISMA flow diagram. (DOC)

S2 File PRISMA checklist. (DOC)

S3 File Meta-analysis on genetic association studies checklist. (DOCX)

S4 File Articles excluded from the meta-analysis. (DOCX)

S5 File Search strategy. (DOCX)
