# Supplementary material for: Association of the IL-1RN variable number of tandem repeat polymorphism and Helicobacter pylori infection: A meta-analysis
Source: PLoS One. 2017 Apr 6;12(4):e0175052. doi: 10.1371/journal.pone.0175052 (PMC5383105; doi:10.1371/journal.pone.0175052)
Supplement: S1 File — (DOC) [file pone.0175052.s001.doc]

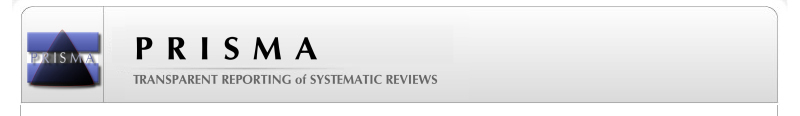
**PRISMA 2009 Flow Diagram**

**Screening**

**Included**

**Eligibility**

**Identification**

Records identified through database searching
(Pubmed= 113, Embase = 131)

Additional records identified through other sources
(n = 0)

Records after duplicates removed
(n = 139)

Records screened
(n = 139)

Records excluded

By title and abstract
(n = 59)

Full-text articles assessed for eligibility
(n = 80)

Full-text articles excluded, without sufficient data
(n = 64)

Studies included in qualitative synthesis
(n = 18)

Studies included in quantitative synthesis (meta-analysis)
(n = 18)

Additional articles included through references
(n = 2)
