# Supplementary material for: Association of the IL-1RN variable number of tandem repeat polymorphism and Helicobacter pylori infection: A meta-analysis
Source: PLoS One. 2017 Apr 6;12(4):e0175052. doi: 10.1371/journal.pone.0175052 (PMC5383105; doi:10.1371/journal.pone.0175052)
Supplement: S4 File — (DOCX) [file pone.0175052.s004.docx]

**Articles excluded from our meta-analysis**

**The following articles were excluded as meta-analysis studies:**

1. Zhang BB, Li Y, Feng JQ, Bian DL, Gao XM, Ran MY. No association between IL-1RN VNTR and the risk of duodenal ulcer: a meta-analysis. Human immunology. 2013;74(9):1170-8. Epub 2013/06/27. doi: 10.1016/j.humimm.2013.06.023. PubMed PMID: 23800434.

2. Zhang Y, Liu C, Peng H, Zhang J, Feng Q. IL1 receptor antagonist gene IL1-RN variable number of tandem repeats polymorphism and cancer risk: a literature review and meta-analysis. PloS one. 2012;7(9):e46017. Epub 2012/10/11. doi: 10.1371/journal.pone.0046017. PubMed PMID: 23049925; PubMed Central PMCID: PMCPmc3457944.

3. Persson C, Canedo P, Machado JC, El-Omar EM, Forman D. Polymorphisms in inflammatory response genes and their association with gastric cancer: A HuGE systematic review and meta-analyses. American journal of epidemiology. 2011;173(3):259-70. Epub 2010/12/24. doi: 10.1093/aje/kwq370. PubMed PMID: 21178102; PubMed Central PMCID: PMCPmc3105271.

4. Sugimoto M, Yamaoka Y, Furuta T. Influences of inflammatory cytokine polymorphisms on eradication rates of H. pylori. Helicobacter. 2010;15(4):342.

5. Peleteiro B, Lunet N, Carrilho C, Durães C, Machado JC, La Vecchia C, et al. Association between cytokine gene polymorphisms and gastric precancerous lesions: Systematic review and meta-analysis. Cancer Epidemiology Biomarkers and Prevention. 2010;19(3):762-76.

6. Gianfagna F, De Feo E, van Duijn CM, Ricciardi G, Boccia S. A systematic review of meta-analyses on gene polymorphisms and gastric cancer risk. Current genomics. 2008;9(6):361-74. Epub 2009/06/10. doi: 10.2174/138920208785699544. PubMed PMID: 19506726; PubMed Central PMCID: PMCPmc2691668.

**The following articles were excluded as reviews or letters:**

7. Jia ZF, Zhang SL, Cao XY, Zhou BS, Jiang J. Interaction between Helicobacter pylori and host genetic variants in gastric carcinogenesis. Future Oncology. 2016;12(18):2127-34.

8. Chiurillo MA. Role of gene polymorphisms in gastric cancer and its precursor lesions: current knowledge and perspectives in Latin American countries. World journal of gastroenterology. 2014;20(16):4503-15. Epub 2014/05/02. doi: 10.3748/wjg.v20.i16.4503. PubMed PMID: 24782603; PubMed Central PMCID: PMCPmc4000487.

9. Correa P. Gastric Cancer. Overview. Gastroenterology Clinics of North America. 2013;42(2):211-7.

10. Micev M, Cosic-Micev M. [Pathology and pathobiology of the gastric carcinoma]. Acta chirurgica Iugoslavica. 2011;58(1):39-52. Epub 2011/06/04. PubMed PMID: 21634103.

11. Perri F, Terracciano F, Gentile M, Merla A, Scimeca D, Zullo A. Role of interleukin polymorphisms in gastric cancer: "Pros and cons". World journal of gastrointestinal oncology. 2010;2(6):265-71. Epub 2010/12/17. doi: 10.4251/wjgo.v2.i6.265. PubMed PMID: 21160639; PubMed Central PMCID: PMCPmc2999189.

12. Lao-Sirieix P, Caldas C, Fitzgerald RC. Genetic predisposition to gastro-oesophageal cancer. Current opinion in genetics & development. 2010;20(3):210-7. Epub 2010/03/30. doi: 10.1016/j.gde.2010.03.002. PubMed PMID: 20347291.

13. Sugimoto M, Furuta T, Yamaoka Y. Influence of inflammatory cytokine polymorphisms on eradication rates of Helicobacter pylori. Journal of gastroenterology and hepatology. 2009;24(11):1725-32. Epub 2010/02/09. doi: 10.1111/j.1440-1746.2009.06047.x. PubMed PMID: 20136959; PubMed Central PMCID: PMCPmc3128255.

14. Peleteiro B, Carrilho C, Modcoicar P, Cunha L, Ismail M, Guisseve A, et al. Chronic atrophic gastritis, intestinal metaplasia, helicobacter pylori virulence, IL1RN polymorphisms, and smoking in dyspeptic patients from Mozambique and Portugal. Helicobacter. 2009;14(4):306-8.

15. Nyerere K, Sayed S, Revathi G, Ojwang P, Matiru V, Devani S, et al. Prevalence of gastric mucosal interleukin-1 polymorphisms in Kenyan patients with advanced gastric cancer. South African medical journal = Suid-Afrikaanse tydskrif vir geneeskunde. 2009;99(2):95-7. Epub 2009/05/08. PubMed PMID: 19418669.

16. Kononov AV. [Genetic regulation and phenotype of inflammation in Helicobacter pylori infection]. Arkhiv patologii. 2009;71(5):57-63. Epub 2009/11/27. PubMed PMID: 19938708.

17. Chourasia D, Ghoshal UC. Pathogenesis of gastro-oesophageal reflux disease: what role do Helicobacter pylori and host genetic factors play? Tropical gastroenterology : official journal of the Digestive Diseases Foundation. 2008;29(1):13-9. Epub 2008/06/21. PubMed PMID: 18564661.

18. Prinz C, Schwendy S, Voland P. H pylori and gastric cancer: shifting the global burden. World journal of gastroenterology. 2006;12(34):5458-64. Epub 2006/09/29. PubMed PMID: 17006981; PubMed Central PMCID: PMCPmc4088226.

19. Basso D, Plebani M. H. pylori infection: bacterial virulence factors and cytokine gene polymorphisms as determinants of infection outcome. Critical reviews in clinical laboratory sciences. 2004;41(3):313-37. Epub 2004/08/17. doi: 10.1080/10408360490472804. PubMed PMID: 15307635.

20. Hamajima N. Persistent Helicobacter pylori infection and genetic polymorphisms of the host. Nagoya journal of medical science. 2003;66(3-4):103-17. Epub 2004/01/20. PubMed PMID: 14727687.

**The following articles were excluded as irrelevant studies:**

21. Tabata N, Hokimoto S, Akasaka T, Sueta D, Arima Y, Sakamoto K, et al. Helicobacter Pylori-seropositivity along with genetic and environmental factors predicts clinical outcome after acute coronary syndrome. International Journal of Cardiology. 2016;212:54-6.

22. Phiphatpatthamaamphan K, Siramolpiwat S, Chonprasertsuk S, Tangaroonsanti A, Pornthisarn B, Bhanthumkomol P, et al. Effect of IL-1 polypmorphisms, CYP2C19 genotype and antibiotic resistance for helicobacter pylori eradication comparing between 10-day sequential therapy and 14-day standard triple therapy with four times daily dosing of amoxicillin in Thailand: A prospective randomized controlled study. Gastroenterology. 2016;150(4):S880.

23. Pittayanon R, Vilaichone RK, Mahachai V, Goh KL. Influences of duration of treatment, CYP2C19 genotyping, interleukin-1 polymorphisms and antibiotic resistant strains in helicobacter pylori eradication rates. Gastroenterology. 2015;148(4):S598-S9.

24. Companioni O, Bonet C, Garcia N, Sanz-Anquela JM, Berdasco M, Adrados M, et al. Significant genetic variability associated with the evolution of gastric cancer precursor lesions in a Spanish population. Gastroenterology. 2015;148(4):S357.

25. Al-Safar H, Kamal W, Hassoun A, Almahmeed W, Rais N. Combined association analysis of interleukin 1-receptor antagonist (IL-1RN) variable number of tandem repeat (VNTR) and Haptoglobin 1/2 polymorphisms with type 2 diabetes mellitus risk. Journal of diabetes and metabolic disorders. 2015;15:10. Epub 2015/01/01. doi: 10.1186/s40200-016-0232-z. PubMed PMID: 27030821; PubMed Central PMCID: PMCPmc4812649.

26. Nurgalieva A, Shaymardanova E, Khidiyatova IM, Nadyrshina DD, Gabbasova LV, Kuramshina OA, et al. [Association of cytokine gene polymorphisms in peptic ulcer development in the Bashkortostan Republic]. Genetika. 2014;50(12):1455-65. Epub 2015/05/16. PubMed PMID: 25975153.

27. Gonzalez-Hormazabal P, Musleh M, Bustamante M, Stambuk J, Escandar S, Valladares H, et al. Host polymorphisms and virulence genotypes of helicobacter pylori as risk factors for gastric cancer. Anticancer Research. 2014;34(10):5927.

28. Yanovich O, Titov L, Nosova E, Doroshko M, DuBuske L. Influence of polymorphisms in inflammatory cytokines on risk of duodenal ulcer among patients with Helicobacter pylori infections. Allergy: European Journal of Allergy and Clinical Immunology. 2011;66:484.

29. Queiroz DM, Rocha GA, Moura SB, Rocha AMC, Cabral MMA, Paim AA, et al. Signal transducer and activator of transcription 3 (STAT 3) gene polymorphism and gastric carcinoma. Gastroenterology. 2011;140(5):S353.

30. Martinez T, Hernandez-Suarez G, Bravo MM, Trujillo E, Quiroga A, Albis R, et al. [Association of interleukin-1 genetic polymorphism and CagA positive Helicobacter pylori with gastric cancer in Colombia]. Revista medica de Chile. 2011;139(10):1313-21. Epub 2012/01/31. doi: /S0034-98872011001000010. PubMed PMID: 22286731.

31. Zhang L, Mei Q, Li QS, Hu YM, Xu JM. The effect of cytochrome P2C19 and interleukin-1 polymorphisms on H. pylori eradication rate of 1-week triple therapy with omeprazole or rabeprazole, amoxycillin and clarithromycin in Chinese people. Journal of clinical pharmacy and therapeutics. 2010;35(6):713-22. Epub 2010/11/09. doi: 10.1111/j.1365-2710.2009.01140.x. PubMed PMID: 21054464.

32. Kononov AV, Pomorgailo EG, Potrokhova EA, Filipenko ML. [Polymorphism of cytokine genes in the development of Helicobacter pylori infection]. Vestnik Rossiiskoi akademii meditsinskikh nauk. 2010;(2):8-12. Epub 2010/04/07. PubMed PMID: 20364673.

33. Hung KH, Hung HW, Yang HB, Lu CC, Wu JJ, Sheu BS. Host single nucleotide polymorphisms of MMP-9 -1562/TIMP-1 372 have gender differences in the risk of gastric intestinal metaplasia after Helicobacter pylori infection. Helicobacter. 2009;14(6):580-7. Epub 2009/11/06. doi: 10.1111/j.1523-5378.2009.00717.x. PubMed PMID: 19889076.

34. Brzezniakiewicz K. [The role of interleukin-1 gene polymorphism in gastric carcinogenesis associated with Helicobacter pylori infection]. Annales Academiae Medicae Stetinensis. 2009;55(3):5-12. Epub 2009/01/01. PubMed PMID: 20698171.

35. Suzuki T, Matsushima M, Shirakura K, Koike J, Masui A, Takagi A, et al. Association of inflammatory cytokine gene polymorphisms with platelet recovery in idiopathic thrombocytopenic purpura patients after the eradication of Helicobacter pylori. Digestion. 2008;77(2):73-8. Epub 2008/03/21. doi: 10.1159/000121392. PubMed PMID: 18354254.

36. Ryberg A, Borch K, Sun YQ, Monstein HJ. Concurrent genotyping of Helicobacter pylori virulence genes and human cytokine SNP sites using whole genome amplified DNA derived from minute amounts of gastric biopsy specimen DNA. BMC microbiology. 2008;8:175. Epub 2008/10/10. doi: 10.1186/1471-2180-8-175. PubMed PMID: 18842150; PubMed Central PMCID: PMCPmc2577186.

37. Erzin Y, Koksal V, Altun S, Dobrucali A, Aslan M, Erdamar S, et al. Role of host interleukin 1beta gene (IL-1B) and interleukin 1 receptor antagonist gene (IL-1RN) polymorphisms in clinical outcomes in Helicobacter pylori-positive Turkish patients with dyspepsia. Journal of gastroenterology. 2008;43(9):705-10. Epub 2008/09/23. doi: 10.1007/s00535-008-2220-7. PubMed PMID: 18807132.

38. Achyut BR, Moorchung N, Mittal B. Genetic association of interleukin-1 haplotypes with gastritis and precancerous lesions in North Indians. Clinical and experimental medicine. 2008;8(1):23-9. Epub 2008/04/04. doi: 10.1007/s10238-008-0152-4. PubMed PMID: 18385937.

39. Abuzarova ER, Gorshkov OV, Chernova OA, Chernov VM, Akberova NI, Abdulkhakov RA. [Peculiarities of genotype distribution of interleukins (IL-1 and IL-10) in patients with peptic ulcer disease and their associations with persistence of Mycoplasma hyorhinis and Helicobacter pylori genotypes]. Eksperimental'naia i klinicheskaia gastroenterologiia = Experimental & clinical gastroenterology. 2008;(6):27-31. Epub 2008/01/01. PubMed PMID: 19334424.

40. Zhang D, Zheng H, Zhou Y, Tang X, Yu B, Li J. Association of IL-1beta gene polymorphism with cachexia from locally advanced gastric cancer. BMC Cancer. 2007;7.

41. Zambon CF, Fasolo M, Basso D, D'Odorico A, Stranges A, Navaglia F, et al. Clarithromycin resistance, tumor necrosis factor alpha gene polymorphism and mucosal inflammation affect H. pylori eradication success. Journal of gastrointestinal surgery : official journal of the Society for Surgery of the Alimentary Tract. 2007;11(11):1506-14; discussion 14. Epub 2007/09/12. doi: 10.1007/s11605-007-0246-4. PubMed PMID: 17846855.

42. Seno H, Satoh K, Tsuji S, Shiratsuchi T, Harada Y, Hamajima N, et al. Novel interleukin-4 and interleukin-1 receptor antagonist gene variations associated with non-cardia gastric cancer in Japan: comprehensive analysis of 207 polymorphisms of 11 cytokine genes. Journal of gastroenterology and hepatology. 2007;22(5):729-37. Epub 2007/04/21. doi: 10.1111/j.1440-1746.2007.04934.x. PubMed PMID: 17444864.

43. Ito H, Kaneko K, Makino R, Konishi K, Kurahashi T, Yamamoto T, et al. Interleukin-1beta gene in esophageal, gastric and colorectal carcinomas. Oncology reports. 2007;18(2):473-81. Epub 2007/07/06. PubMed PMID: 17611673.

44. Sugimoto M, Furuta T, Shirai N, Ikuma M, Hishida A, Ishizaki T. Influences of proinflammatory and anti-inflammatory cytokine polymorphisms on eradication rates of clarithromycin-sensitive strains of Helicobacter pylori by triple therapy. Clinical pharmacology and therapeutics. 2006;80(1):41-50. Epub 2006/07/04. doi: 10.1016/j.clpt.2006.03.007. PubMed PMID: 16815316.

45. Starzynska T, Ferenc K, Wex T, Kahne T, Lubinski J, Lawniczak M, et al. The association between the interleukin-1 polymorphisms and gastric cancer risk depends on the family history of gastric carcinoma in the study population. The American journal of gastroenterology. 2006;101(2):248-54. Epub 2006/02/04. doi: 10.1111/j.1572-0241.2006.00422.x. PubMed PMID: 16454826.

46. Shirai K, Ohmiya N, Taguchi A, Mabuchi N, Yatsuya H, Itoh A, et al. Interleukin-8 gene polymorphism associated with susceptibility to non-cardia gastric carcinoma with microsatellite instability. Journal of gastroenterology and hepatology. 2006;21(7):1129-35. Epub 2006/07/11. doi: 10.1111/j.1440-1746.2006.04443.x. PubMed PMID: 16824064.

47. Al-Moundhri MS, Al-Nabhani M, Al-Bahrani B, Burney IA, Al-Madhani A, Ganguly SS, et al. Interleukin-1beta gene (IL-1B) and interleukin 1 receptor antagonist gene (IL-1RN) polymorphisms and gastric cancer risk in an Omani Arab population. Gastric cancer : official journal of the International Gastric Cancer Association and the Japanese Gastric Cancer Association. 2006;9(4):284-90. Epub 2007/01/20. doi: 10.1007/s10120-006-0392-5. PubMed PMID: 17235630.

48. Ruzzo A, Graziano F, Pizzagalli F, Santini D, Battistelli V, Panunzi S, et al. Interleukin 1B gene (IL-1B) and interleukin 1 receptor antagonist gene (IL-1RN) polymorphisms in Helicobacter pylori-negative gastric cancer of intestinal and diffuse histotype. Annals of oncology : official journal of the European Society for Medical Oncology. 2005;16(6):887-92. Epub 2005/04/27. doi: 10.1093/annonc/mdi184. PubMed PMID: 15851404.

49. Perri F, Piepoli A, Bonvicini C, Gentile A, Quitadamo M, Di Candia M, et al. Cytokine gene polymorphisms in gastric cancer patients from two Italian areas at high and low cancer prevalence. Cytokine. 2005;30(5):293-302.

50. Palli D, Saieva C, Luzzi I, Masala G, Topa S, Sera F, et al. Interleukin-1 gene polymorphisms and gastric cancer risk in a high-risk Italian population. The American journal of gastroenterology. 2005;100(9):1941-8. Epub 2005/09/01. doi: 10.1111/j.1572-0241.2005.50084.x. PubMed PMID: 16128937.

51. Wu MS, Shun CT, Huang SP, Cheng AL, Chen LT, Lin JT. Effect of interleukin-1β and glutathione S-transferase genotypes on the development of gastric mucosa-associated lymphoid tissue lymphoma. Haematologica. 2004;89(8):1015-7.

52. Rad R, Dossumbekova A, Neu B, Lang R, Bauer S, Saur D, et al. Cytokine gene polymorphisms influence mucosal cytokine expression, gastric inflammation, and host specific colonisation during Helicobacter pylori infection. Gut. 2004;53(8):1082-9. Epub 2004/07/13. doi: 10.1136/gut.2003.029736. PubMed PMID: 15247172; PubMed Central PMCID: PMCPmc1774164.

53. Kang WK, Park WS, Chin HM, Park CH. [The role of interleukin-1beta gene polymorphism in the gastric carcinogenesis]. The Korean journal of gastroenterology = Taehan Sohwagi Hakhoe chi. 2004;44(1):25-33. Epub 2004/07/22. PubMed PMID: 15266130.

54. Matsuo K, Hamajima N, Ikehara Y, Suzuki T, Nakamura T, Matsuura A, et al. Smoking and polymorphisms of fucosyltransferase gene Le affect success of H. pylori eradication with lansoprazole, amoxicillin, and clarithromycin. Epidemiology and infection. 2003;130(2):227-33. Epub 2003/05/06. PubMed PMID: 12729191; PubMed Central PMCID: PMCPmc2869958.

55. Garza-Gonzalez E, Hold G, Perez-Perez GI, Bosques-Padilla FJ, Tijerina-Menchaca R, Maldonado-Garza HJ, et al. [Role of polymorphism of certain cytokines in gastric cancer in Mexico. Preliminary results]. Revista de gastroenterologia de Mexico. 2003;68(2):107-12. Epub 2004/05/07. PubMed PMID: 15127646.

56. Zambon CF, Basso D, Navaglia F, Germano G, Gallo N, Milazzo M, et al. Helicobacter pylori virulence genes and host IL-1RN and IL-1beta genes interplay in favouring the development of peptic ulcer and intestinal metaplasia. Cytokine. 2002;18(5):242-51. Epub 2002/08/06. PubMed PMID: 12161099.

57. Lin RC, Morris BJ. Association analysis of polymorphisms at the interleukin-1 locus in essential hypertension. American journal of medical genetics. 2002;107(4):311-6. Epub 2002/02/13. PubMed PMID: 11840488.

58. Hwang IR, Kodama T, Kikuchi S, Sakai K, Peterson LE, Graham DY, et al. Effect of interleukin 1 polymorphisms on gastric mucosal interleukin 1beta production in Helicobacter pylori infection. Gastroenterology. 2002;123(6):1793-803. Epub 2002/11/28. doi: 10.1053/gast.2002.37043. PubMed PMID: 12454835.

59. Figueiredo C, Machado JC, Pharoah P, Seruca R, Sousa S, Carvalho R, et al. Helicobacter pylori and interleukin 1 genotyping: an opportunity to identify high-risk individuals for gastric carcinoma. Journal of the National Cancer Institute. 2002;94(22):1680-7. Epub 2002/11/21. PubMed PMID: 12441323.

**The following articles were excluded with insufficient data：**

60. Ramis IB, Vianna JS, Halicki PC, Lara C, Tadiotto TF, da Silva Maciel JB, et al. Relationship of interleukin-1B gene promoter region polymorphism with Helicobacter pylori infection and gastritis. J Infect Dev Ctries. 2015; 9(10):1108-16. Epub 2015/11/01. doi: 10.3855/jidc.6123. PubMed PMID: 26517486.

61. Martínez T, Hernández GA, Bravo MM, Trujillo E, Pérez-García J, Robayo JC, et al. Pre-cancerous gastric lesions in Colombian patients: Association with IL-1B-511, IL-1RN, IL-10-919, IL-10-1082, TNF-α-308 genes polymorphisms, and anti-Helicobacter pylori cagA IgG antibodies. Revista Colombiana de Cancerologia. 2014; 18(1):8-17.

62. Akcil G, Dogan I, Cengiz M, Engin ED, Dogan M, Unal S, et al. The role of interleukin-1 gene polymorphisms and Helicobacter pylori in gastroesophageal reflux disease. Turk J Gastroenterol. 2014; 25 Suppl 1:81-5. Epub 2015/04/25. doi: 10.5152/tjg.2014.6512. PubMed PMID: 25910374.

63. Ryberg A, Petersson F, Redeen S, Eriksson O, Borch K. Host Gene Polymorphisms in Relation to Helicobacter Pylori Infection and Associated Diseases in a Population Based Cohort. Gastroenterology research. 2013; 6(6):207-18. Epub 2013/12/01. doi: 10.4021/gr578w. PubMed PMID: 27785255; PubMed Central PMCID: PMCPmc5051128.

64. Mattar R, Marques SB, Dos Santos AF, do Socorro Monteiro M, Iriya K, Carrilho FJ. A possible role of IL-1RN gene polymorphism in the outcome of gastrointestinal diseases associated with H. pylori infection. Clin Exp Gastroenterol. 2013; 6:35-41. Epub 2013/05/03. doi: 10.2147/ceg.s42260. PubMed PMID: 23637547; PubMed Central PMCID: PMCPmc3634316.

65. Marcos-Pinto R, Dinis-Ribeiro M, Carneiro F, Wen X, Lopes C, Figueiredo C, et al. First-degree relatives of early-onset gastric cancer patients show a high risk for gastric cancer: phenotype and genotype profile. Virchows Archiv: an international journal of pathology. 2013; 463(3):391-9. Epub 2013/07/28. doi: 10.1007/s00428-013-1458-5. PubMed PMID: 23887584.

66. Kim JJ, Kim N, Hwang S, Kim JY, Kim JY, Choi YJ, et al. Relationship of interleukin-1beta levels and gastroesophageal reflux disease in Korea. J Gastroenterol Hepatol. 2013;28(1):90-8. Epub 2012/10/02. doi: 10.1111/j.1440-1746.2012.07274.x. PubMed PMID: 23020284.

67. da Costa DM, Neves-Filho EH, Alves MK, Rabenhorst SH. Interleukin polymorphisms and differential methylation status in gastric cancer: an association with Helicobacter pylori infection. Epigenomics. 2013;5(2):167-75. Epub 2013/04/10. doi: 10.2217/epi.13.7. PubMed PMID: 23566094.

68. Oliveira JG, Duarte MC, Silva AE. IL-1ra anti-inflammatory cytokine polymorphism is associated with risk of gastric cancer and chronic gastritis in a Brazilian population, but the TNF-beta pro-inflammatory cytokine is not. Mol Biol Rep. 2012;39(7):7617-25. Epub 2012/02/14. doi: 10.1007/s11033-012-1596-x. PubMed PMID: 22327782.

69. Marcos-Pinto R, Dinis-Ribeiro MJ, Carneiro F, Machado JC, Figueiredo C, Wen X, et al. High-risk host genotype and H. Pylori strains in first degree relatives of patients with early-onset gastric cancer. Gastroenterology. 2012;142(5):S627-S8.

70. Sugimoto M, Yamaoka Y, Furuta T. Influences of inflammatory cytokine polymorphisms on eradication rates of Helicobacter pylori. Gastroenterology. 2011;140(5):S881.

71. Shin CM, Kim N, Lee HS, Lee DH, Kim JS, Jung HC, et al. Intrafamilial aggregation of gastric cancer: a comprehensive approach including environmental factors, Helicobacter pylori virulence, and genetic susceptibility. European journal of gastroenterology & hepatology. 2011;23(5):411-7. Epub 2011/04/20. doi: 10.1097/MEG.0b013e328343b7f5. PubMed PMID: 21502924.

72. Li J, Wang F, Zhou Q, Ou Z, Jia H, Deng X, et al. IL-1 polymorphisms in children with peptic symptoms in South China. Helicobacter. 2011;16(3):246-51. Epub 2011/05/19. doi: 10.1111/j.1523-5378.2011.00837.x. PubMed PMID: 21585612.

73. Titov LP, Yanovich OO, Nosova ES, Doroshko MV, DuBuske LM. Association of gastric disease due to helicobacter pylori with polymorphisms in interleukin-1 receptor antagonist (IL-1RN) and TNF-alpha genes in Belarus. Annals of Allergy, Asthma and Immunology. 2010;105(5):A93-A4.

74. Sugimoto M, Yamaoka Y, Furuta T. Influence of interleukin polymorphisms on development of gastric cancer and peptic ulcer. World J Gastroenterol. 2010;16(10):1188-200. Epub 2010/03/12. PubMed PMID: 20222161; PubMed Central PMCID: PMCPmc2839170.

75. Mei Q, Xu JM, Cao HL, Bao DM, Hu NZ, Zhang L, et al. Associations of the IL-1 and TNF gene polymorphisms in the susceptibility to duodenal ulcer disease in Chinese Han population. Int J Immunogenet. 2010;37(1):9-12. Epub 2009/10/07. doi: 10.1111/j.1744-313X.2009.00882.x. PubMed PMID: 19804405.

76. Chiurillo MA, Moran Y, Canas M, Valderrama E, Alvarez A, Armanie E. Combination of Helicobacter pylori-iceA2 and proinflammatory interleukin-1 polymorphisms is associated with the severity of histological changes in Venezuelan chronic gastritis patients. FEMS Immunol Med Microbiol. 2010;59(2):170-6. Epub 2010/05/21. doi: 10.1111/j.1574-695X.2010.00675.x. PubMed PMID: 20482626.

77. Abuzarova E, Chernova O, Shaimardanova G, Gorshkov O, Abdulkhakov R, Chernov V. Peculiarities of gastric mucosa epithelium ultrastructure in patients with H. pyloriassociated gastric ulcer disease with different polymorphic loci of cytokine genes (IL-1 and IL-10) genotypes. Helicobacter. 2010;15(4):348.

78. Shtygasheva OV, Ageeva ES, Ryazantseva NV. Polymorphisms in genes interleukins associated with ulcer disease in a high-risk Khakas population. Helicobacter. 2009;14(4):359.

79. Queiroz DM, Rocha AMC, Melo FF, Rocha GA, Souza C, Saraiva IS, et al. The platelet count increase associated with H. pylori eradication is accomplished by a reversal of TH1 cytokine profile. Gastroenterology. 2009;136(5):A123.

80. Murphy G, Thornton J, McManus R, Swan N, Ryan B, Hughes DJ, et al. Association of gastric disease with polymorphisms in the inflammatory-related genes IL-1B, IL-1RN, IL-10, TNF and TLR4. European journal of gastroenterology & hepatology. 2009;21(6):630-5. Epub 2009/03/20. doi: 10.1097/MEG.0b013e3283140eea. PubMed PMID: 19295440; PubMed Central PMCID: PMCPmc2802816.

81. Melo Barbosa HP, Martins LC, Dos Santos SE, Demachki S, Assumpcao MB, Aragao CD, et al. Interleukin-1 and TNF-alpha polymorphisms and Helicobacter pylori in a Brazilian Amazon population. World J Gastroenterol. 2009;15(12):1465-71. Epub 2009/03/27. PubMed PMID: 19322919; PubMed Central PMCID: PMCPmc2665140.

82. Gao L, Weck MN, Nieters A, Brenner H. Inverse association between a pro-inflammatory genetic profile and Helicobacter pylori seropositivity among patients with chronic atrophic gastritis: enhanced elimination of the infection during disease progression? European journal of cancer (Oxford, England : 1990). 2009;45(16):2860-6. Epub 2009/05/12. doi: 10.1016/j.ejca.2009.04.015. PubMed PMID: 19427781.

83. Con SA, Takeuchi H, Con-Chin GR, Con-Chin VG, Yasuda N, Con-Wong R. Role of bacterial and genetic factors in gastric cancer in Costa Rica. World J Gastroenterol. 2009;15(2):211-8. Epub 2009/01/10. PubMed PMID: 19132772; PubMed Central PMCID: PMCPmc2653314.

84. Chourasia D, Achyut BR, Tripathi S, Mittal B, Mittal RD, Ghoshal UC. Genotypic and functional roles of IL-1B and IL-1RN on the risk of gastroesophageal reflux disease: the presence of IL-1B-511*T/IL-1RN*1 (T1) haplotype may protect against the disease. The American journal of gastroenterology. 2009;104(11):2704-13. Epub 2009/07/16. doi: 10.1038/ajg.2009.382. PubMed PMID: 19603010.

85. Barbosa HPM, Martins LC, dos Santos SEB, Demachki S, Assumpção MB, Aragão CD, et al. Interleukin-1 and TNF-α polymorphisms and Helicobacter pylori in a Brazilian Amazon population. World Journal of Gastroenterology. 2009;15(12):1465-71.

86. Abuzarova E, Chernova O, Gorshkov O, Abdulkhakov R, Chernov V. Distribution of H. pylori genotypes, polymorphic loci of cytokine genes (IL-1 and IL-10), and ulcer sizes in patients with duodenal ulcer disease in Kazan, Russia. Helicobacter. 2009;14(4):356.

87. Sierra R, Une C, Ramirez V, Alpizar-Alpizar W, Gonzalez MI, Ramirez JA, et al. Relation of atrophic gastritis with Helicobacter pylori-CagA(+) and interleukin-1 gene polymorphisms. World J Gastroenterol. 2008;14(42):6481-7. Epub 2008/11/26. PubMed PMID: 19030199; PubMed Central PMCID: PMCPmc2773333.

88. Shin WG, Jang JS, Kim HS, Kim SJ, Kim KH, Jang MK, et al. Polymorphisms of interleukin-1 and interleukin-2 genes in patients with gastric cancer in Korea. J Gastroenterol Hepatol. 2008;23(10):1567-73. Epub 2008/09/03. doi: 10.1111/j.1440-1746.2008.05479.x. PubMed PMID: 18761558.

89. Kim N, Park YS, Cho SI, Lee HS, Choe G, Kim IW, et al. Prevalence and risk factors of atrophic gastritis and intestinal metaplasia in a Korean population without significant gastroduodenal disease. Helicobacter. 2008;13(4):245-55. Epub 2008/07/31. doi: 10.1111/j.1523-5378.2008.00604.x. PubMed PMID: 18665932.

90. Feng Y, Zhang J, Dai L, Zhang J, Wang P, Zang J, et al. Inflammatory cytokine gene polymorphisms in gastric cancer cases' and controls' family members from Chinese areas at high cancer prevalence. Cancer Lett. 2008;270(2):250-9. Epub 2008/06/21. doi: 10.1016/j.canlet.2008.05.013. PubMed PMID: 18565646.

91. Crusius JB, Canzian F, Capella G, Pena AS, Pera G, Sala N, et al. Cytokine gene polymorphisms and the risk of adenocarcinoma of the stomach in the European prospective investigation into cancer and nutrition (EPIC-EURGAST). Annals of oncology : official journal of the European Society for Medical Oncology. 2008;19(11):1894-902. Epub 2008/07/17. doi: 10.1093/annonc/mdn400. PubMed PMID: 18628242.

92. Achyut BR, Moorchung N, Srivastava AN, Gupta NK, Mittal B. Risk of lymphoid follicle development in patients with chronic antral gastritis: role of endoscopic features, histopathological parameters, CagA status and interleukin-1 gene polymorphisms. Inflammation research : official journal of the European Histamine Research Society [et al]. 2008;57(2):51-6. Epub 2008/02/22. doi: 10.1007/s00011-007-7033-2. PubMed PMID: 18288454.

93. Liou JM, Lin JT, Wang HP, Huang SP, Lee YC, Chiu HM, et al. IL-1B-511 C→T polymorphism is associated with increased host susceptibility to Helicobacter pylori infection in Chinese. Helicobacter. 2007;12(2):142-9.

94. Garcia-Gonzalez MA, Lanas A, Quintero E, Nicolas D, Parra-Blanco A, Strunk M, et al. Gastric cancer susceptibility is not linked to pro-and anti-inflammatory cytokine gene polymorphisms in whites: a Nationwide Multicenter Study in Spain. The American journal of gastroenterology. 2007;102(9):1878-92. Epub 2007/07/21. doi: 10.1111/j.1572-0241.2007.01423.x. PubMed PMID: 17640324.

95. Con SA, Con-Wong R, Con-Chin GR, Con-Chin VG, Takeuchi H, Valerin AL, et al. Serum pepsinogen levels, Helicobacter pylori CagA Status, and cytokine gene polymorphisms associated with gastric premalignant lesions in Costa Rica. Cancer epidemiology, biomarkers & prevention : a publication of the American Association for Cancer Research, cosponsored by the American Society of Preventive Oncology. 2007;16(12):2631-6. Epub 2007/12/19. doi: 10.1158/1055-9965.epi-07-0215. PubMed PMID: 18086767.

96. Zabaleta J, Camargo MC, Piazuelo MB, Fontham E, Schneider BG, Sicinschi LA, et al. Association of interleukin-1beta gene polymorphisms with precancerous gastric lesions in African Americans and Caucasians. The American journal of gastroenterology. 2006;101(1):163-71. Epub 2006/01/13. doi: 10.1111/j.1572-0241.2006.00387.x. PubMed PMID: 16405550.

97. Sicinschi LA, Lopez-Carrillo L, Camargo MC, Correa P, Sierra RA, Henry RR, et al. Gastric cancer risk in a Mexican population: role of Helicobacter pylori CagA positive infection and polymorphisms in interleukin-1 and -10 genes. Int J Cancer. 2006;118(3):649-57. Epub 2005/08/23. doi: 10.1002/ijc.21364. PubMed PMID: 16114018.

98. Morgan DR, Dominguez RL, Keku TO, Heidt PE, Martin CF, Galanko JA, et al. Gastric cancer and the high combination prevalence of host cytokine genotypes and Helicobacter pylori in Honduras. Clinical gastroenterology and hepatology : the official clinical practice journal of the American Gastroenterological Association. 2006;4(9):1103-11. Epub 2006/07/06. doi: 10.1016/j.cgh.2006.05.025. PubMed PMID: 16820326.

99. Leung WK, Chan MC, To KF, Man EP, Ng EK, Chu ES, et al. H. pylori genotypes and cytokine gene polymorphisms influence the development of gastric intestinal metaplasia in a Chinese population. The American journal of gastroenterology. 2006;101(4):714-20. Epub 2006/04/26. doi: 10.1111/j.1572-0241.2006.00560.x. PubMed PMID: 16635219.

100. Zumkeller N, Koenig W, Hoffmann MM, Kolb H, Brenner H, Rothenbacher D. Helicobacter pylori seropositive subjects do not show a pronounced systemic inflammatory response even in the presence of the interleukin-1 receptor antagonist gene polymorphism. Epidemiol Infect. 2005;133(3):569-72. Epub 2005/06/21. PubMed PMID: 15962564; PubMed Central PMCID: PMCPmc2870281.

101. Zambon CF, Basso D, Navaglia F, Belluco C, Falda A, Fogar P, et al. Pro- and anti-inflammatory cytokines gene polymorphisms and Helicobacter pylori infection: interactions influence outcome. Cytokine. 2005;29(4):141-52. Epub 2005/01/18. doi: 10.1016/j.cyto.2004.10.013. PubMed PMID: 15652446.

102. Taguchi A, Ohmiya N, Shirai K, Mabuchi N, Itoh A, Hirooka Y, et al. Interleukin-8 promoter polymorphism increases the risk of atrophic gastritis and gastric cancer in Japan. Cancer epidemiology, biomarkers & prevention : a publication of the American Association for Cancer Research, cosponsored by the American Society of Preventive Oncology. 2005;14(11 Pt 1):2487-93. Epub 2005/11/15. doi: 10.1158/1055-9965.epi-05-0326. PubMed PMID: 16284368.

103. Rocha GA, Guerra JB, Rocha AM, Saraiva IE, da Silva DA, de Oliveira CA, et al. IL1RN polymorphic gene and cagA-positive status independently increase the risk of noncardia gastric carcinoma. Int J Cancer. 2005;115(5):678-83. Epub 2005/02/11. doi: 10.1002/ijc.20935. PubMed PMID: 15704154.

104. Queiroz DM, Bittencourt P, Guerra JB, Rocha AM, Rocha GA, Carvalho AS. IL1RN polymorphism and cagA-positive Helicobacter pylori strains increase the risk of duodenal ulcer in children. Pediatr Res. 2005;58(5):892-6. Epub 2005/09/27. doi: 10.1203/01.pdr.0000181380.14230.8b. PubMed PMID: 16183821.

105. Lu W, Pan K, Zhang L, Lin D, Miao X, You W. Genetic polymorphisms of interleukin (IL)-1B, IL-1RN, IL-8, IL-10 and tumor necrosis factor {alpha} and risk of gastric cancer in a Chinese population. Carcinogenesis. 2005;26(3):631-6. Epub 2004/12/08. doi: 10.1093/carcin/bgh349. PubMed PMID: 15579481.

106. Hellmig S, Titz A, Steinel S, Ott S, Folsch UR, Hampe J, et al. Influence of IL-1 gene cluster polymorphisms on the development of H. pylori associated gastric ulcer. Immunol Lett. 2005;100(2):107-12. Epub 2005/05/12. doi: 10.1016/j.imlet.2005.04.001. PubMed PMID: 15885804.

107. Garza-Gonzalez E, Bosques-Padilla FJ, El-Omar E, Hold G, Tijerina-Menchaca R, Maldonado-Garza HJ, et al. Role of the polymorphic IL-1B, IL-1RN and TNF-A genes in distal gastric cancer in Mexico. Int J Cancer. 2005;114(2):237-41. Epub 2004/11/13. doi: 10.1002/ijc.20718. PubMed PMID: 15540224.

108. Garcia-Gonzalez MA, Savelkoul PH, Benito R, Santolaria S, Crusius JB, Pena AS, et al. No allelic variant associations of the IL-1 and TNF gene polymorphisms in the susceptibility to duodenal ulcer disease. Int J Immunogenet. 2005;32(5):299-306. Epub 2005/09/17. doi: 10.1111/j.1744-313X.2005.00528.x. PubMed PMID: 16164697.

109. Chang YW, Jang JY, Kim NH, Lee JW, Lee HJ, Jung WW, et al. Interleukin-1B (IL-1B) polymorphisms and gastric mucosal levels of IL-1beta cytokine in Korean patients with gastric cancer. Int J Cancer. 2005;114(3):465-71. Epub 2004/11/20. doi: 10.1002/ijc.20724. PubMed PMID: 15551344.

110. Alpizar-Alpizar W, Perez-Perez GI, Une C, Cuenca P, Sierra R. Association of interleukin-1B and interleukin-1RN polymorphisms with gastric cancer in a high-risk population of Costa Rica. Clin Exp Med. 2005;5(4):169-76. Epub 2005/12/20. doi: 10.1007/s10238-005-0082-3. PubMed PMID: 16362796.

111. Yang J, Hu ZB, Xu YC, Shen J, Niu JY, Hu X, et al. Association between polymorphisms of interleukin-1B and interleukin-1 receptor antagonist genes and host susceptibility to gastric cancer. World Chinese Journal of Digestology. 2004;12(8):1769-73.

112. Queiroz DM, Guerra JB, Rocha GA, Rocha AM, Santos A, De Oliveira AG, et al. IL1B and IL1RN polymorphic genes and Helicobacter pylori cagA strains decrease the risk of reflux esophagitis. Gastroenterology. 2004;127(1):73-9. Epub 2004/07/06. PubMed PMID: 15236174.

113. Lobo Gatti L, Rodríguez Burbano R, Pimentel De Assumpção P, De Arruda Cardoso Smith M, Marques Payão SL. Interleukin-1β polymorphisms, Helicobacter pylori infection in individuals from Northern Brazil with gastric adenocarcinoma. Clinical and Experimental Medicine. 2004;4(2):93-8.

114. Hsu PI, Li CN, Tseng HH, Lai KH, Hsu PN, Lo GH, et al. The interleukin-1 RN polymorphism and Helicobacter pylori infection in the development of duodenal ulcer. Helicobacter. 2004;9(6):605-13. Epub 2004/12/22. doi: 10.1111/j.1083-4389.2004.00277.x. PubMed PMID: 15610073.

115. Glas J, Torok HP, Schneider A, Brunnler G, Kopp R, Albert ED, et al. Allele 2 of the interleukin-1 receptor antagonist gene is associated with early gastric cancer. Journal of clinical oncology : official journal of the American Society of Clinical Oncology. 2004;22(23):4746-52. Epub 2004/12/01. doi: 10.1200/jco.2004.03.034. PubMed PMID: 15570075.

116. Chen A, Li CN, Hsu PI, Lai KH, Tseng HH, Hsu PN, et al. Risks of interleukin-1 genetic polymorphisms and Helicobacter pylori infection in the development of gastric cancer. Alimentary pharmacology & therapeutics. 2004;20(2):203-11. Epub 2004/07/06. doi: 10.1111/j.1365-2036.2004.01826.x. PubMed PMID: 15233701.

117. Zeng ZR, Hu PJ, Hu S, Pang RP, Chen MH, Ng M, et al. Association of interleukin 1B gene polymorphism and gastric cancers in high and low prevalence regions in China. Gut. 2003;52(12):1684-9. Epub 2003/11/25. PubMed PMID: 14633943; PubMed Central PMCID: PMCPmc1773879.

118. Rad R, Prinz C, Neu B, Neuhofer M, Zeitner M, Voland P, et al. Synergistic effect of Helicobacter pylori virulence factors and interleukin-1 polymorphisms for the development of severe histological changes in the gastric mucosa. The Journal of infectious diseases. 2003;188(2):272-81. Epub 2003/07/11. doi: 10.1086/376458. PubMed PMID: 12854083.

119. Machado JC, Figueiredo C, Canedo P, Pharoah P, Carvalho R, Nabais S, et al. A proinflammatory genetic profile increases the risk for chronic atrophic gastritis and gastric carcinoma. Gastroenterology. 2003;125(2):364-71. Epub 2003/08/02. PubMed PMID: 12891537.

120. Koivurova OP, Karhukorpi JM, Joensuu ET, Koistinen PO, Valtonen JM, Karttunen TJ, et al. IL-1 RN 2/2 genotype and simultaneous carriage of genotypes IL-1 RN 2/2 and IL-1beta-511 T/T associated with oesophagitis in Helicobacter pylori-negative patients. Scand J Gastroenterol. 2003;38(12):1217-22. Epub 2004/01/31. PubMed PMID: 14750640.

121. Garcia-Gonzalez MA, Lanas A, Savelkoul PH, Santolaria S, Benito R, Crusius JB, et al. Association of interleukin 1 gene family polymorphisms with duodenal ulcer disease. Clin Exp Immunol. 2003;134(3):525-31. Epub 2003/11/25. PubMed PMID: 14632761; PubMed Central PMCID: PMCPmc1808893.

122. Chang YT, Wu MS, Shun CT, Lin MT, Chang MC, Lin JT. Association of polymorphisms of interleukin-1 beta gene and Helicobacter pylori infection with the risk of gastric ulcer. Hepatogastroenterology. 2002;49(47):1474-6. Epub 2002/09/21. PubMed PMID: 12239970.

123. Garcia-Gonzalez MA, Lanas A, Santolaria S, Crusius JB, Serrano MT, Pena AS. The polymorphic IL-1B and IL-1RN genes in the aetiopathogenesis of peptic ulcer. Clin Exp Immunol. 2001;125(3):368-75. Epub 2001/09/05. PubMed PMID: 11531943; PubMed Central PMCID: PMCPmc1906147.
