# Supplementary material for: Association of the IL-1RN variable number of tandem repeat polymorphism and Helicobacter pylori infection: A meta-analysis
Source: PLoS One. 2017 Apr 6;12(4):e0175052. doi: 10.1371/journal.pone.0175052 (PMC5383105; doi:10.1371/journal.pone.0175052)
Supplement: S5 File — (DOCX) [file pone.0175052.s005.docx]

**PubMed**

#1 "interleukin-1 receptor antagonist"[Title/Abstract] OR "IL-1Ra"[Title/Abstract] OR "IL1ra"[Title/Abstract] OR "interleukin-1RN"[Title/Abstract] OR "IL1RN"[Title/Abstract] OR "IL-1RN"[Title/Abstract]

#2 "polymorphism"[Title/Abstract] OR "polymorphisms"[Title/Abstract] OR "SNP"[Title/Abstract] OR "variant"[Title/Abstract]

#3 "*Helicobacter pylori*"[Title/Abstract] OR "*H pylori*"[Title/Abstract] OR "HP"[Title/Abstract] OR "*Helicobacter*"[Title/Abstract]

#4 #1 AND #2 AND #3
